# Cellular kinases incorporated into HIV-1 particles: passive or active passengers?

**DOI:** 10.1186/1742-4690-8-71

**Published:** 2011-09-02

**Authors:** Charline Giroud, Nathalie Chazal, Laurence Briant

**Affiliations:** 1 Centre d'études d'agents Pathogènes et Biotechnologies pour la Santé (CPBS), UMR5236 CNRS - Université Montpellier 1-Montpellier 2, Montpellier, France

## Abstract

Phosphorylation is one of the major mechanisms by which the activities of protein factors can be regulated. Such regulation impacts multiple key-functions of mammalian cells, including signal transduction, nucleo-cytoplasmic shuttling, macromolecular complexes assembly, DNA binding and regulation of enzymatic activities to name a few. To ensure their capacities to replicate and propagate efficiently in their hosts, viruses may rely on the phosphorylation of viral proteins to assist diverse steps of their life cycle. It has been known for several decades that particles from diverse virus families contain some protein kinase activity. While large DNA viruses generally encode for viral kinases, RNA viruses and more precisely retroviruses have acquired the capacity to hijack the signaling machinery of the host cell and to embark cellular kinases when budding. Such property was demonstrated for HIV-1 more than a decade ago. This review summarizes the knowledge acquired in the field of HIV-1-associated kinases and discusses their possible function in the retroviral life cycle.

## Review

The genetic information of human immunodeficiency virus type 1 (HIV-1) is carried by an RNA genome of approximately 9.3 Kb packaged into viral particles as a non-covalent dimer [1]. This genetic material contains nine open reading frames that encode fifteen proteins, including structural proteins (matrix, capsid, nucleocapsid and p6), envelope glycoproteins (gp120 and gp41) and enzymes (protease, reverse transcriptase and integrase). Six additional open reading frames direct the synthesis of accessory and regulatory proteins (Tat, Rev, Nef, Vpr, Vpu and Vif). These proteins have complex functions and generally interface with the host cell machinery. The mature structural proteins and enzymes, Vpr, Nef and Vif are contained in a spherical particle surrounded by a lipid bilayer acquired from the host cell plasma membrane containing the envelope glycoproteins. Early after HIV-1 discovery, the particle was also proven to package components from the host cell. The first studies performed using classical biochemistry together with more recent analysis relying on systematic mass spectrometry sequencing have inventoried the presence of a wide variety of cellular proteins in highly purified HIV-1 virions [2,3]. While a fraction have been reported to be required for viral infectivity, a proportion of these components appear to be non-essential for replication in a new target cell. The presence of cellular proteins with varying functional importance in viral particles may reflect differences in the mechanisms accounting for the viral incorporation of these host factors. Indeed, at later replication stages, HIV-1-encoded proteins are directed to the site of assembly and form a bud consisting of cellular membranes and cytoplasm. This particular step favors the passive incorporation into HIV-1 virions of host cell factors constitutively located at the plasma membrane or present in the cytosol beneath the budding bilayer. Alternatively, the budding particle incorporates cellular factors attracted to the assembly site through specific interactions with viral components. This last model is particularly illustrated by the packaging of cofactors assisting late retroviral replication, including proteins from trafficking systems ensuring targeting of viral proteins and nucleic acids to the budding site, cofactors required for viral assembly and cellular complexes involved in the budding and release of the retroviral particle. An informative approach to differentiate between these two classes of virus-associated cell factors was proposed by Hammarstedt and Garoff [4]. By measuring the concentrations of cellular proteins relative to the lipid content in the viral particles and in the membranes of donor cells expressing or not HIV-1 proteins, they discriminated between the number of factors selectively enriched in the viral particle and those passively packaged into HIV-1. Using this strategy, the increased concentration of cyclophilin A and Tsg101 observed at the plasma membrane upon Gag precursor expression suggested a selective recruitment into viruses rather than a passive incorporation. On the contrary, actin and clathrin appeared to "diffuse" into virions because their respective concentrations at the membrane remained unchanged regardless of whether Gag was expressed. Considering the cellular proteins selectively attracted into HIV-1 particles through interactions with viral proteins or nucleic acids, their requirement for propagation in a new target cell is also variable. Indeed, a number of packaged cell factors, including those recruited to support late replication, have been assumed to be non-essential to the early intracellular steps of replication. Conversely, some cellular proteins that have no proven role in late replication, and are actively recruited into HIV-1, are strictly required for propagation in a new target cell. A number of these components have been shown to assist the intracellular steps of future infectious cycles that are not fully ensured by the HIV-1-encoded proteins. Encapsidation and subsequent delivery of these proteins to the target cell are supposed either to compensate for the lack of essential cellular cofactors or to render them available at the site which supports replication even if expressed in the cell. An interesting approach to question the functional importance of packaged cellular proteins is to investigate their capacity to be incorporated into particles of HIV-1-related viruses. Comparative studies showed that several proteins already found to be packaged into HIV-1 particles through specific interactions with viral proteins or nucleic acids are also detected in HIV-2 and in simian immunodeficiency virus (SIV) particles (see Table 1 for references). For some proteins (discussed below), their conservation was extended to more distant retroviruses, such as HTLV-1. The significance of such similarities is questionable. It may be either argued for the conservation of a common mechanism of replication throughout viral evolution, or it may be considered as a proof for the non-specific association of proteins with distantly related viruses. In a number of cases, including for some kinases, evidence for the conservation of interaction motives in viral proteins together with functional studies of viruses unable to package these cellular factors proved that these components retain an evolutionarily conserved function [5-9].

**Table 1:** Virion-associated cellular proteins in HIV-1, HIV-2 and SIV

Cellular proteins	Virus			
		
		HIV-1	HIV-2	SIV
Chaperone	Hsp70	+ [32]	+ [32]	+ [32]
	Cyclophilin A	+ [26]	- [26,124]	-/+^a ^[26,124]
	Pin 1	+ [37]	*ND*	+ [5]
				
Cytoskeleton	Actin	+ [37]	*ND*	+ [5]
	Moesin	+ [37]	*ND*	+ [5]
	Ezrin	+ [37]	*ND*	*ND*
	Arp2/3	+ [37]	*ND*	+ [5]
				
Vacuolar sorting	Tsg101	+ [83]	+ [125]	*ND*
	Alix	+ [84]	*ND*	+ [84]
	Ubiquitin	+ [37]	*ND*	+ [126]
				
Nucleic acids binding	UNG2	+ [47]	- [127]	- [127]
	APOBEC3G	+ [46]	*ND*	+ [128]
	Staufen	+ [43]	+ [43]	+ [43]
	INI1/HSNF5	+ [42]	- [42]	- [42]
				
Kinases	ERK2	+ [6,7]	*ND*	+ [5]
	PKA	+ [14]	*ND*	+ [5]

^a ^incorporated in some SIV strains (SIV_cpz_)

*ND*: not determined.

Giroud *et al. *Table 1

## Experimental strategies for the identification of HIV-1-associated proteins

In addition to the difficulty associated with discriminating between host factors that are selectively or passively packaged into viruses, the identification of cellular proteins embedded in viral particles is technically complicated. The most critical aspect is the strict necessity to discriminate between virus-incorporated components and cellular factors contaminating viral preparations. The latter group includes proteins docked to the outside of cell-free virions. This group also comprises cellular proteins contained in microvesicles and exosomes with sizes and densities comparable to viruses, that co-sediment with viral preparations and represent a source of contamination even after the density gradient separation of viral particles [3,10,11]. Accordingly, sample preparation should be performed carefully. Two reference protocols have been developed to produce preparations of highly purified HIV-1 [12]. One approach involves the digestion of viral samples using the non-specific serine protease subtilisin. Subtilisin digestion of HIV-1 preparations eliminates more than 95% of the microvesicle-associated proteins and removes contaminants docked to the outside of viruses. The effectiveness of this protocol is determined by the size reduction of the gp41 transmembrane envelope glycoprotein. This method is particularly adapted to the study of proteins inside the virions. Alternatively, CD45 immunoaffinity depletion of HIV-1 can be used to isolate viruses from cellular exosomes. This technique, which was developed based on the observation that CD45 membrane molecules are discarded from HIV-1 viruses produced from hematopoietic cells [13], has been previously combined with mass spectrometry analysis to produce an impressive list of cell factors packaged into HIV-1 particles produced from primary macrophages [2,3]. CD45 depletion is most useful for studies that require the exterior of the virions to be intact. In any case, electron microscopy imaging provides a reliable method to discriminate between assembled viruses and exosomes from a morphological point of view and to validate the presence of host proteins in virions, as previously reported [14].

Another technical feature to consider when studying HIV-1-associated cellular factors is the cell type and the viral isolate or strain used to prepare the biological samples. The array of packaged cellular proteins may differ greatly according to the host cell used to propagate the virus. This aspect has been well documented for membrane molecules embedded in the envelope of virions produced from various T cell lines. The acquisition of CD55 and CD59 complement decay factors [15], LFA-1 [16] and MHC class I and II molecules [16,17] is host-dependent. Distinct incorporation profiles have also been reported when viruses produced from permanent cell lines or primary peripheral blood mononuclear cells were analyzed [15,18]. Regarding cytosolic proteins, this point has not been comprehensively studied. However, LC-MS/MS analysis of viruses grown on macrophages [3] failed to detect the presence of some components that were identified from viruses grown on T lymphocytes. Notably, ERK-2, a kinase detected from the HIV-1_HZ321 _isolate grown in HUT78 T lymphocytes [6] and from HIV-1_ELI _viruses prepared from MT4 cells [7], was not detected from HIV-1_NLAD8 _grown in primary macrophages [3]. The functional significance for such difference remains unknown, and its correlation with the biology of HIV-1 replication in distinct cell types remains to be analyzed. Nevertheless, the nature of cellular proteins packaged in HIV-1 needs to be discussed according to the method in which the viruses are purified, the nature of the viral strain and the cell type used for viral production.

## Main families of cytoplasmic proteins detected in HIV-1 virions

The above mentioned strategies have led to the identification of a surprisingly large variety of HIV-1 associated cellular components (referenced in the web-based database http://web.ncifcrf.gov/research/avp/), among which only a small fraction has been functionally characterized. A significant proportion of these molecules are glycoproteins expressed at the surface of the host cell that become incorporated into the lipid bilayer surrounding the retroviral particle, as extensively reviewed previously [19]. Upon encountering their natural ligand at the surface of the target cell, they contribute to the initiation and stabilization of the virus-cell contact [20-24], and in some cases stimulate signaling cascades and various cellular responses (e.g., inflammation, apoptosis and the modulation of immune responses) [13,25]. Regarding proteins of cytosolic and nuclear origins, the list of cellular factors associated with HIV-1 is particularly impressive [3]. Without providing an exhaustive inventory, some families of proteins have been highlighted.

### Cellular chaperones are abundantly incorporated into HIV-1

The HIV-1-associated protein that was the most extensively studied is certainly the peptidyl-prolyl isomerase cyclophilin A. Cyclophilin A was detected early as an essential component for the viral core organization [26]. Approximately 200 molecules are incorporated into one viral particle, and its interaction with the p24 capsid protein determines viral infectivity [27,28]. Other proteins from the chaperone family have been detected in HIV-1 viruses, including heat shock proteins Hsp40, Hsp60, Hsp90 and Hsp70, and the Pin1 peptidyl-prolyl cis/trans isomerase [29-31]. The function of HIV-1 associated chaperones appears to be generally related to the regulation of capsid organization, as cyclophilin A, Hsp70 and Pin1 have been proposed to be involved in core reorganization during assembly and post-entry events [32,33]. This function is not the only one ascribed to these proteins, particularly Pin 1. Indeed, Pin1 directly interacts with the antiviral cytidine deaminase Apobec3G and reduces its incorporation into viruses [34]. In addition, Pin1 exerts a stabilizing effect on the retroviral integrase by catalyzing conformational modifications of the enzyme and promotes HIV-1 genome integration in primary CD4^+ ^T lymphocytes [35]. These functions were attributed to Pin1 in HIV-1 infected cells. Despite the fact that the contribution of the virus-associated protein in these last two functions remains to be investigated, it is conceivable that the presence of Pin1 inside viral particles could assist early replication by first counteracting residual Apobec3G proteins, which could escape Vif degradation and be incorporated into HIV-1 and second by stimulating viral integration.

### Proteins from trafficking systems

Proteins participating in the trafficking systems of endogenous cargoes are packaged within HIV-1 viruses. This group includes an important variety of components and regulators of the cytoskeleton network (actin protein, Arp2/3, HS-1, ezrin, moesin and cofilin) [28,31,36,37]. This group also comprises components of microtubules (tubulin subunits and the hexokinase-3 molecular motor [3]). In addition, a series of proteins that participate in the vesicular trafficking machinery (Tsg101, Alix, Vps28, Vps4A, Tal and free ubiquitin) [2,3,38] and host factors required for vesicular transport (notably LAMP1 and SNARE) [3] and endocytosis (Rab5a [3], vATPase [3], and clathrin [39]) are packaged into viruses. The presence of these components is thought to reflect the hijacking of the cell trafficking machinery when viral components are transported to the assembly site. Interestingly, actin and moesin, in addition to unrelated cell-derived proteins, such as EF-1α and NDR1/2 (discussed below), have been detected in HIV-1 virions as cleavage products [31,40]. Experiments conducted using defective HIV-1 mutants suggested that these proteins are digested by the retroviral protease. However, the functional significance for the processing of HIV-1-associated cellular proteins has not been elucidated.

### Nuclear proteins incorporated into HIV-1 particles

The viral incorporation of nuclear proteins is typically illustrated by the selective packaging of histones (H4, H2B and H3.1) and a number of proteins that interact with nucleic acids. This group comprises the active histone deacetylase HDAC1 and the chromatin remodeling protein INI1/HSNF5, which is selectively incorporated into HIV-1 virions (but excluded from other retroviral particles) [41,42]. This group also includes the double-stranded RNA-binding protein Staufen 1 [43], which supports viral assembly and is packaged through interactions with HIV-1 genomic RNA and the nucleocapsid domain of Gag [44]. Finally, a number of nucleic acid-modifying and -repairing enzymes are also detected in HIV-1 particles. The cytidine deaminase Apobec3G is incorporated into Vif-depleted HIV-1 viruses [45]. In wild-type HIV-1, the incorporation of Apobec3G is counteracted by Vif through the help of the proteasome degradation system [46]. Because Apobec3G has demonstrated anti-viral effects [46], HIV-1 is thought to have acquired the capacity to encode proteins counteracting the incorporation of cellular factors detrimental to replication when they are packaged into virions. The packaging of uracil DNA glycosylase 2 (UNG2), a DNA-repair enzyme required for the excision of uracil misincorporated into genomic DNA, also illustrates the capacity of HIV-1 to incorporate nuclear proteins from the host cell [47]. However, the function of HIV-1-associated UNG2 remains controversial [48]. This protein was alternatively proposed to assist the reverse transcriptase and to control uracilation of the neoysnthesized proviral DNA [49], to be dispensable for HIV-1 replication [50], or to favor the degradation of the Apobec3G-edited HIV-1 provirus [51]. Interestingly, expression of cellular UNG2 is dramatically decreased by HIV-1 Vpr, theoretically preventing UNG2 packaging at high levels [52,53]. As UNG2 was reported to display antiviral activities [51,53], Vpr-mediated degradation could be considered as a defense mechanism developed to control activity of an antiviral factor likely to be incorporated into HIV-1.

## Protein kinases packaged into HIV-1

In this review, we focused primarily on the class of HIV-1-associated host cytosolic factors known as protein kinases. Phosphorylation is one of the major mechanisms through which the activity of protein factors can be regulated. In mammalian cells, up to 30% of all proteins may be modified by phosphorylation. Such regulation impacts multiple levels, including nucleo-cytoplasmic shuttling, the assembly of macromolecular complexes, DNA-binding capacity and enzymatic activation. The presence of kinase activities in viral particles was observed early. The first observations in the field demonstrated that high levels of protein kinase activity are packaged in the Rauscher murine leukemia virus [54] and vaccinia virus [55], and established that the product of the transforming Rous sarcoma virus exhibits phosphotransferase capacities [56]. Since these observations, widespread interest in the study of virus/kinases relationships has developed. In a significant number of models, primarily large DNA viruses (such as *Herpesviridae*, *Poxviridae*, *Baculoviridae*), the phosphorylation of viral proteins can be catalyzed by protein kinases encoded by the viral genome. The knowledge in this field has recently been summarized in a complete review [57]. Regarding HIV-1 and related retroviruses, the viral genome is devoid of genes encoding protein kinase. The presence of intraviral kinase activity is strictly related to its capacity to package cellular enzymes into its particles (see Table 2). This aspect of HIV-1 biology remains poorly understood. Indeed, while a significant number of studies performed during the past decade have determined the capacity of HIV-1 to activate cellular kinases, particularly following binding of the viral envelope to its receptors or subsequently to intracellular replication [58-60], little attention has been devoted to the characterization of virus-associated kinases and to the study of their functional roles. To date, a small number of tyrosine or serine-threonine kinases from cellular origin have been reported to be embedded in HIV-1. Some have received poor attention, such as p56^*lck*^, cdc42, PKC and STAT1 for which only their presence in the virus has been reported [3,31]. The kinases that have received the most interest are ERK2, PKA and NDR1/2 kinases. The knowledge accumulated regarding their functions and their incorporation into budding structures is discussed below.

**Table 2:** Virion-associated cellular protein kinases and their viral substrates

Family	Genus	Virus	Viral substrate(s)	Virus-associated cellular kinase (s)	Possible function(s)	References
Retroviridae	Alpharetrovirus	AMV	(?)	42-46 kDa and 60-64 kDa kinases	10 to 25 kDa viral protein	[54,129,130]
		RSV	(?)	Cellular kinase (?)	(?)	[56]
	Betaretrovirus	MMTV	(?)	Cellular kinase (?)	(?)	[56]
	Gammaretrovirus	MSV	(?)	Cellular kinase (?)	(?)	[131-133]
		R-MLV	(?)	42-46 kDa and 60-64 kDa Kinases	(?)	[54,129,130]
		FeLV	(?)	Cellular kinase (?)	(?)	[56]
	Deltaretrovirus	HTLV-1	MA	ERK2	Virus assembly & release	[6,8]
	Lentivirus	HIV-1	CA, MA, p6	ERK2, PKA, DR1/2, 53 kDa (?),	Virus infectivity, uncoating	[3,6,7,14,40]
			Rev, Nef, Vif	p56^*lck*^, PKC, PRP2, Nm23-H1	Virus release, replication (?)	[62-64,82,88,85]
		SIV	(?)	ERK2, PKA, PKC	(?)	[5,76]
		FIV	(?)	ERK2	(?)	[76]

Giroud *et al. *Table 2

### ERK2 (HIV-1 produced from lymphoblastoid cell lines; method of detection: biochemical subtilisin resistance)

The MAPKinase ERK2 was the first protein kinase of cellular origin to be detected within HIV-1 viruses [6,7]. The viral packaging of this protein has been evidenced by biochemical detection of ERK2 in ultra-purified preparation of virions and was further confirmed by phosphorylation assays performed using a viral lysate as a source of kinase [6,7]. Its functional role has finally been addressed by the study of HIV-1 particles produced by cell either cultured in the presence chemical inhibitors that interfere directly with ERK2 activation or expressing dominant negative forms of ERK2 upstream activators Ras, Raf or MEK1 [7,61]. This strategy showed that HIV-1 particles devoid of ERK2 activity are poorly infectious. Such viruses are unable to complete reverse transcription of the viral genome. They produce reduced levels of strong-stop DNA, indicating that the virus-associated kinase is required for an early step of infection. To date, the exact function of ERK2 packaged in HIV-1 remains unclear. A number of studies pointed to the capacity of the kinase to phosphorylate HIV-1 proteins including Rev [61], Nef [62] and Vif [63,64]. The contribution of ERK2 in the functional role of Rev and Nef remains incompletely clarified. Regarding Vif, despite its function was initially proposed to be regulated by ERK2 mediated phosphorylation [63], ERK2 has latter been reported to enhance replication in Vif-independent cell lines [61]. Accordingly, ERK2's contribution in viral infectivity has been proposed to be in some extent independent to its capacity to phosphorylate Vif. Moreover, for all three proteins, the contribution of the packaged isoform of the kinase remains far from demonstrated.

**Figure 1:**
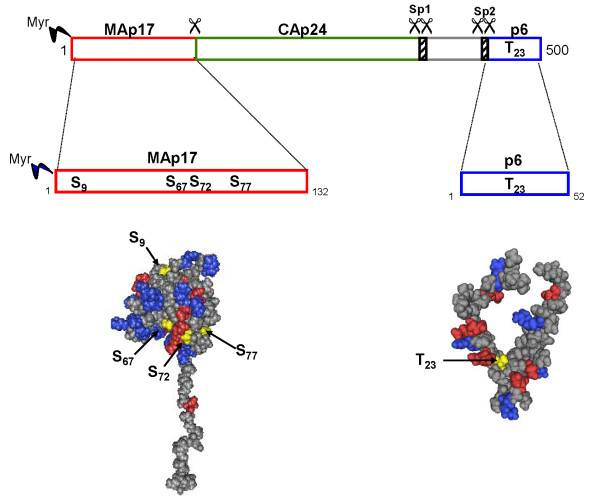
**Schematic representation of HIV-1 Gag and 3D structure of the matrix and p6 proteins**. Positions of S_9_, S_67_, S_72 _and S_77 _residues are indicated in scheme and positioned in three-dimensional structure of the HIV-1 matrix protein [119] (MMDB ID: 53369). Position of T_23 _is indicated in scheme and positioned in structure of HIV-1 p6 protein [120] (MMDB ID: 36264).

Attempts to identify the function of HIV-1-associated ERK2 have rather focused on its capacity to phosphorylate the retroviral matrix protein (MA) [7]. A fraction of MA molecules is phosphorylated in infected cells [65]. Analyzing the functional role of these phosphorylation events has generated extensive controversy [66-68]. MA is involved in multiple steps of the HIV-1 replication cycle. It has been proposed to direct viral proteins trafficking via nuclear import and export functions [69]. More specifically, MA directs targeting of the preintegration complexes to the nucleus during the early phase of infection. This function relies on the presence of two nuclear localization signals in MA [70]. Interestingly, MA has been reported to localize more predominantly at the plasma membrane of infected cells when viruses display reduced ERK2 activity [65]. Consistent with this model, alanine substitution of four highly conserved serine residues at positions 9, 67, 72 and 77, which had been identified as major phosphoacceptor amino acids in MA (Figure 1), blocked HIV-1 replication at a post-entry step of infection in permanent cell lines and non-dividing macrophages [65,71]. The possibility that global MA phosphorylation unmasks a nuclear localization signal has been previously proposed for tyrosine phosphorylation in MA [67]. However, because single serine to alanine substitution of the above mentioned conserved residues does not markedly influence HIV-1 replication and has no effect on the MA N-terminal myristate exposure [72], it has rather been suggested that the additive effect of serine phosphorylation in MA increases the negative charge of the molecule and promotes the electrostatic repulsion between clusters of positively charged residues in MA and the inner layer of the plasma membrane [65]. In contradiction with these data, some studies reported that the distribution of phosphorylated MA mirrors the total MA within the cell [73,74]. Altogether these results suggest that phosphorylation alone does not result in a shift of MA from a membrane-bound to a membrane-free state. While no consensus has been reached regarding the precise function of ERK2-mediated MA phosphorylation, new information on the possible function of the virus-associated kinase during early HIV-1 replication has been recently published. Based on previous evidence for the contribution of emerin, a constituent of the inner nuclear envelope, in the nuclear translocation and integration of HIV-1 provirus into chromosomes of cell cycle-arrested primary T lymphocytes and macrophages [75], a recent study demonstrated that HIV-1-associated ERK2 activity, the ERK2-dependent phosphorylation of emerin and viral DNA integration are intimately correlated [76]. According to these results, the phosphorylation of emerin by encapsidated ERK2 would promote the chromatin engagement of HIV-1 provirus. However, the requirement for emerin in HIV-1 infectivity is controversial [77,78]. Moreover, while the study by Bukong et al. clearly demonstrated that the phosphorylation of emerin can contribute to processes leading to proviral integration, the respective contribution of cellular ERK2 and that of the virus-associated kinase remains to be formally defined. Indeed, these results were produced using VSV envelope glycoprotein-pseudotyped HIV-1, which is unable to trigger physiological activation signals in the host cell. We and others have demonstrated that the attachment of the HIV-1 envelope to its cellular receptors activates the MAPKinase signaling pathway in which ERK2 participates [79-81]. Accordingly, repeating these experiments with viruses containing an HIV-1 envelope would help to decipher the relative physiological role of cellular versus virus-packaged ERK2 in the nuclear import and the post-nuclear steps of HIV-1 replication.

Finally, searching for ERK2 substrates has evidenced that the kinase also phosphorylates the L-domain-containing p6 protein [82]. The completion of retroviral budding requires the recruitment of cellular proteins associated with the endocytic machinery, namely ESCRT complexes. For HIV-1, this function is fulfilled by the p6 domain in Gag polyprotein precursor. Canonical PTAP and YXXLF sequences located at the N-terminus and C-terminus ends of p6, respectively, are required for interaction with Tsg101 and Alix/AIP-1 proteins in the ESCRT1 complex [83,84]. The Tsg101/p6 interaction is optimized by p6 monoubiquitination and a perturbation by mean of proteasome inhibitors profoundly interferes with viral release, morphology and infectivity of secreted virions [83,84]. Together with H. G. Krausslich's group, we have reported that the HIV-1 p6 protein is phosphorylated by ERK2 both in the context of a Gag polyprotein precursor and of a mature protein packaged within HIV-1 virions [82,85]. We have observed that alanine substitution of the unique phosphoacceptor threonine residue identified in p6 significantly reduces viral particles release and results in the accumulation of immature virions at the plasma membrane of the host cell [82], a phenotype very similar to that reported by others for mutations inhibiting ESCRT1/p6 interactions [83,86]. In contrast, mimicking p6 phosphorylation through an aspartic acid substitution at this site increases the accumulation of mature viruses in intracytoplasmic vacuoles of the producing cell (L.B., personal communication). Accordingly, the ERK2-mediated phosphorylation of p6 may participate in accurate virus-cell membrane separation and proper viral maturation. Because phosphorylation can regulate the recognition of target proteins by ubiquitin-conjugating enzymes, it can be hypothesized that ERK2 could regulate HIV-1 late-budding activities by modifying the recruitment of the vesicular sorting machinery by p6. Interestingly, these observations can be extended to HTLV-1. Indeed, HTLV-1 budding also relies on the recruitment of the ESCRT complexes, which is mediated through interacting motifs located in the MA protein sequence. We have demonstrated that HTLV-1 MA is phosphorylated by ERK2 [8]. As observed for HIV-1, phosphorylation of the L-domain containing protein is required to regulate HLTV-1 particle assembly and release. Despite the fact that ERK2 has been equally detected in purified HTLV-1 and HIV-1 particles [6,82], it is conceivable that in both viral models, the phosphorylation of L-domain-containing proteins is rather mediated by the cellular form of ERK2 rather than by the virus-associated isoform of the kinase.

In summary, the data accumulated since the initial description of ERK2 in HIV-1 particles indicate that the encapsidation of this cellular kinase is strictly required for optimal infectivity. Although its function is not clearly elucidated, virus-associated ERK2 could assist early steps of HIV-1 replication either by supporting the establishment of a functional reverse transcription complex or by regulating nuclear import of the preintegration complexes. Very recent data have shown that ERK2 interacts with the poly-proline motif located near the cyclophilin binding loop at the N-terminus of HIV-1 CA domain of Gag [9]. This motif, conserved in distinct retroviruses, including in all subtypes of HIV-1, HIV-2, SIV, HTLV-I, HTLV-2 and other retroviruses, could account for the evolutionarily conserved incorporation of ERK2 in lentiviruses [5-8]. Characterization of this interaction motif opens a new avenue to investigate the role virus-associated-ERK2, both in the retroviral cycle and in HIV-1-induced pathogenesis.

### PKA (HIV-1 produced from lymphoblastoid cell lines; method of detection: biochemical subtilisin resistance)

**Figure 2:**
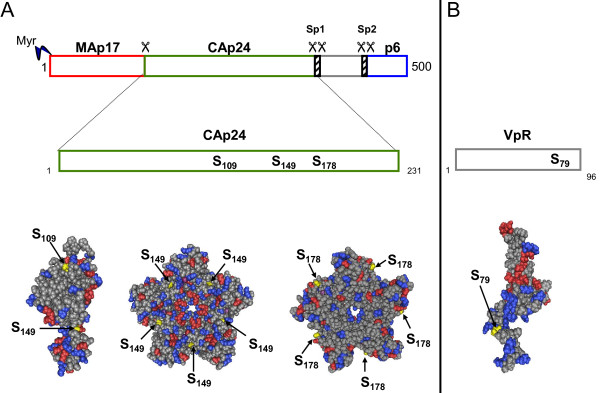
**Schematic representations of Gag, capsid protein and VpR protein of HIV-1**. Positions of S_109_, S_149 _residues are indicated in scheme and positioned in X-Ray structure of HIV-1 capsid protein [121] (MMDB ID: 73892). Positions of S_149_, S_178_, residues are indicated in scheme and positioned in X-Ray structure of pentameric HIV-1 capsid protein [121,122] (MMDB ID: 87889). Position of S_79 _is indicated in scheme and positioned in NMR structure of HIV-1 VpR protein [123] (MMDB ID: 22329).

A second well-documented example of a cellular kinase packaged within HIV-1 particles is the cAMP-dependent protein kinase (PKA). In recent years, our group has demonstrated that PKA is packaged into HIV-1 viruses [14]. In the cell, PKA is found at the plasma membrane or associated with subcellular organelles, and it is anchored to these sites though interactions with AKAP-anchoring proteins (for review see [87]). In its resting state, the kinase generally consists of two regulatory subunits and two catalytic subunits. Upon activation, conformational reorganization generated at the level of the regulatory subunits favors the release of the active kinase. In HIV-1 particles, sole catalytic subunits of PKA have been detected [14]. In agreement with this observation, the lysate of purified HIV-1 viruses displays a kinase activity specific for PKA substrates. Viruses produced from PKA-deficient cell lines are not infectious [14]. In searching for a possible contribution of the kinase in phosphorylating proteins that are incorporated into the viral particle, we have found that PKA interacts with and phosphorylates the p24 capsid protein [14]. Three serine residues in p24 sequence have been identified as phosphoacceptor sites [88] (Figure 2A). To analyze the possible role of PKA-dependent phosphorylation of p24, we have produced HIV-1 mutants unable to undergo phosphorylation, by alanine substitution at each phosphoacceptor site. These mutants are impaired for reverse transcription as observed for PKA-depleted HIV-1. Moreover, the assembly and stability of the corresponding capsids are dramatically impaired [88,89].

**Figure 3:**
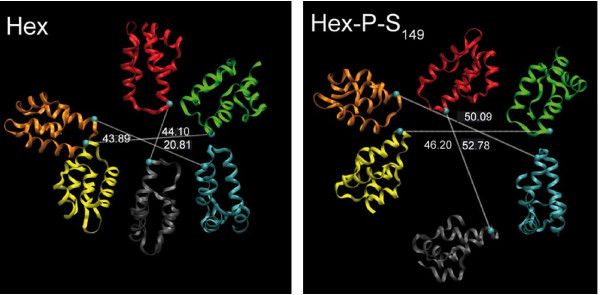
**Molecular dynamics simulations of unphosphorylated and phosphorylated CA hexamers**. Changes observed in the internal diameter of the CTD of the non phosphorylated (left) and S_149_-phosphorylated CA hexamer (right) at 4500 ps. For clarity, only the CTD is represented in ribbons and one color is assigned per monomer [90].

In mature HIV-1 particles, monomeric p24 assembles into a lattice of hexameric and pentameric rings to form a conical core containing the retroviral genome and associated proteins. Our approach developed *in silico *to model the consequences of phosphorylation at the level of a p24 hexamer has revealed that negative charges generated by phosphate conjugation at each serine position favors inter-monomer repulsion or cleavage of important inter-monomeric bonds required for preserving the stability of the hexameric ring of p24 [90] (Figure 3). According to these results, p24 phosphorylation may be considered as an event that could modify organization of p24 hexamers and at a higher order impact the organization of the viral core edifice. This model has been validated using *in vitro *assembly experiments of recombinant p24 with serine-to-aspartic acid mutations, mimicking constitutive phosphorylation. These results need to be considered in the context of the reversible processes required for the association of the retroviral core during the assembly of the viral particle and for the dissociation of the conical capsid once delivered into the target cell. Indeed, phosphorylation and dephosphorylation events are regulators of protein-protein interactions.

Data accumulated from various models have proven that kinases from viral or cellular origins act as regulators of the assembly and disassembly of viral particles by modulating the association or repulsion of proteins involved in the structures of the viral cores and in the packaging of viral genomes. This mechanism is particularly well documented for herpesviruses. Herpes simplex virus type 1 (HSV-1) tegument protein undergoes phosphorylation and dephosphorylation according to the stage of replication. Moreover, the solubility of the viral tegument is significantly enhanced in the presence of ATP-Mg and functional kinase activity [91-94]. In this model, the dissociation of major tegument proteins in infected cells is supposed to be initiated by phosphorylation events mediated both by the UL-13 virus-encoded serine threonine kinase and by cellular kinases [95]. Additional evidence for a role of phosphorylation in the packaging and release of viral nucleic acids has been provided from other viral models, including members of the *Hepadnaviridae *family. The dynamic proteomic study of mature and immature duck hepatitis B virus (DHBV) particles has revealed that cell-associated capsid proteins are highly phosphorylated, while capsids assembled into cell-free virions are dephosphorylated [96,97]. In this model, core protein variants, in which serine acceptor residues have been jointly mutated, display a reduced capacity for nucleic acid encapsidation [98]. From the molecular point of view, hydrogen bonds formed by non-phosphorylated serine have been proposed to stabilize the quaternary structure of DHBV nucleocapsids during assembly. Disruption of these bonds, via subsequent serine phosphorylation, allows the release of genomic DNA from capsids during the early stages of viral infection. Similar mechanisms have been reported for the related human hepatitis B virus (HBV) [99-102]. These events may involve cellular kinases, such as PKC and PKA, and RAP ribosome-associated kinases, which are packaged within the HBV core [103-105]. Finally, this model can be extended to *Togaviridae*. In rubella virus, cycles of alternate phosphorylation of the capsid protein, during the early stages of replication, and dephosphorylation during the latter stages, timely regulate the assembly of the nucleocapsid and the packaging of genomic RNA [106]. Therefore, sequential phosphorylation clearly appears to regulate the ordered progression of viral assembly and disassembly in a number of viral models. In light of the information available on the packaging of active PKA into HIV-1 particles and on its contribution to p24 phosphorylation, this virus-associated kinase may be considered as a possible regulator acting at the level of core organization.

In addition to its contribution in p24 phosphorylation, other functions could potentially be ensured by HIV-1-associated PKA. Indeed, other proteins, including the regulatory proteins Nef and Vpr, have been identified as substrates for the kinase. A single serine residue located at the N-terminus of Nef is phosphorylated by PKA *in vivo *(Figure 2B). Mutation of this residue abrogates the capacity of Nef to enhance HIV-1 replication in unstimulated primary cells [107]. Similarly, PKA triggers the phosphorylation of a single serine residue at position 79 in Vpr. This modification is strictly required for Vpr-dependent cell cycle arrest [108]. Because both Vpr and Nef are embedded in the viral particle, it is conceivable that this phosphorylation may involve the virus-associated PKA kinase. However, at this time, no experimental data is available to validate this hypothesis.

### Nuclear Dbf2-related kinases (NDR) (Epithelial and lymphoid cell lines; method of detection: biochemical subtilisin resistance)

A third family of kinases packaged into HIV virions consists of Dbf2-related kinases. The nuclear NDR1 and cytoplasmic NDR2 Dbf2-related kinases participate in the regulation of cell division and morphology. NDR1/2 kinases remain associated with centrosomal structures throughout the entire cell cycle and regulate their duplication [109]. While endogenous NDR1 has been detected in HIV-1 preparations, the presence of the NDR2 isoform could be seen in viral particles only when the tagged kinase was overexpressed in the virus-producing cell, because of the lack of appropriate detection tools [40]. Interestingly, in HIV-1-infected cells, NDR1 and NDR2 are cleaved at the C-terminus by the retroviral protease. These cleaved isoforms are preferentially packaged into viral particles. The capacity of these cleaved kinases to phosphorylate HIV-1 proteins remains unknown. In addition, the consequences of this proteolytic processing on signaling pathways, controlled by NDR1/2 in the infected cell, have yet to be investigated. The observation that truncated isoforms of NDR kinases relocalize to the nucleus in infected cells provides strong support for the selective recruitment of these proteins into viral particles. The mechanisms underlying the recruitment of cleaved NDR1/2 to the viral particle and the way HIV-1 takes advantage of the packaged NDR1/2 kinases to assist in viral replication yet remain to be determined.

### Other kinases incorporated in HIV-1 virions

In addition to ERK2, PKA and NDR1/2, additional kinases have been detected in purified virions using immunoblotting approaches, high-pressure liquid chromatography, and mass spectrometry analysis. Some kinases remain unidentified, such as a 53 kDa auto-phosphorylable protein that retains serine/threonine kinase activity and was proposed to target the p24 capsid protein [88]. The apparent molecular weights of the NDR kinases rendered possible that they might account for the presence of the anonymous p53 protein in HIV-1. However, the capacity of NDR1/2 to phosphorylate the viral capsid has not been investigated. Among these additional kinases are also protein kinase C [3], phosphoglycerate kinase 1 [3] and p56^*lck *^tyrosine kinase [31] that was proposed to assist HIV assembly at the plasma membrane in its cellular forms [110]. Finally, Nm23-H1 nucleoside diphosphate kinase A [31], a member of the cytoplasmic SET complex with multiple activities, including histidine kinase activity, is also incorporated into HIV-1. Although the function of most of these proteins in HIV-1 replication has yet to be studied, it is interesting to note that Nm23-H1 has been shown to protect HIV-1 and the related viruses HIV-2 and SIV from auto-integration during acute infections when it is expressed in the infected cell [111]. This function could be conserved for the packaged isoforms detected in HIV-1 virions and assist the retroviral replication.

## Future directions in the study of HIV-1-associated kinases

In this review, we have summarized the current knowledge on kinases packaged into HIV-1 and related retroviruses and on their potential substrates. If data accumulated clearly argue for the necessity to incorporate cellular kinases into HIV-1 particles to assist essential steps of the retroviral life cycle, the complete understanding of the functional roles played by virus-associated kinases will require developing new and relevant strategies. Approaches used so far are based on the study either of kinase-deficient viruses or on the characterization of mutant viruses encoding for proteins unable to undergo phosphorylation. As illustrated above, the production of viruses depleted of cellular kinase activities has been generally achieved using chemical inhibitors of cellular kinases added to the culture medium of virus-producing cells. However, although these drugs are effective inhibitors of enzymatic activity, they may have only temporary effects and generally display poor specificity. Overall, the main difficulty in this strategy lies in the fact that these inhibitors are not discriminative solely for the kinase molecules incorporated into viruses. Thus, each study, relying on an inhibitor-based approach, must consider that the drug may not only abolish the virus-associated enzymatic activity, but also potentially interfere with the cellular pool of kinases assisting in intracellular steps of the replicative cycle required to produce infectious viral particles. As a result, the assembly, maturation and organization of the viral particles produced from kinase-deficient cells need to be carefully investigated to guarantee that the phenotype observed for the viruses is strictly linked to the absence of virus-associated kinase activity and not to any side effect. This aspect has been particularly controlled in the study of PKA-deficient viruses [14]. A possible alternative strategy includes the inhibition of protein-protein or protein-nucleic acid interactions underlying the incorporation of cellular kinases into the viral particle. Accordingly, elucidating the mechanisms required for kinase packaging deserves more attention.

The second strategy used in this field relies on the identification of viral substrates modified by the packaged kinases and on the study of viral replication once their phosphorylation sites are mutated. The most common approach relies on the generation of alanine mutants unable to undergo phosphorylation or mutants with acidic (aspartic acid or glutamic acid) substitutions mimicking constitutive phosphorylation of potential target sites. Although it may be informative, this approach fixes the experimental system into a phosphorylated or unphosphorylated state and accounts neither for dynamic phosphorylation and dephosphorylation events nor for the stoichiometry of the reaction. Accordingly, each model elaborated using this strategy needs to be refined to account for the real proportion of molecules phosphorylated *in vivo*. One interesting point is that, in addition to kinases, HIV-1 incorporates additional cellular proteins that naturally counteract phosphate-conjugation activities. Notably, PKC inhibitors and protein phosphatases have been found to be associated with viral particles [3]. These data reinforce the idea that phosphorylation and dephosphorylation are key regulators of the HIV-1 replicative cycle and indicate the capacity of the virus to hijack components of the cell signaling systems to fulfill appropriate functions at the site of replication.

Finally, the above mentioned studies point to the necessity to question the physiological role played by the packaged kinases. Recently, a proteomic analysis of SIV virions revealed that ERK2, PKC and PKA catalytic subunit are packaged into particles [112]. Moreover, this study established that ERK2 is differentially incorporated into viruses produced from disease-resistant sooty mangabeys and disease-susceptible rhesus macaques. This observation strongly argues for a possible role of some packaged kinases in retrovirus pathogenicity and together with functional studies, suggests their potential interest as targets for innovative antivirals. The importance of HIV-1-associated cellular proteins as therapeutic targets has been suggested more than a decade ago. Antibodies targeting surface antigens incorporated in the retroviral bilayer (LFA-1, HLA class I and β2 microglobulin) efficiently block HIV-1 replication [36,112-115]. The interest in such a strategy was recently reassessed using LFA1-1 agonists that potentiated T20 antiviral activity and reduced virus propagation by decreasing cell-to-cell interactions [116]. Inhibition of cellular kinase activities as a strategy to inhibit intracellular steps of HIV-1 replication has been considered and is currently under investigation. Much attention has been focused on several purine derivatives such as roscovitine inhibiting cyclin-dependent kinases required for RNA-polymerase II dependent transcription of integrated viral genes [117]. These molecules have been reported to inhibit HIV-1 *in vitro *and *in vivo *with limited emergence of resistance [118]. If used to abolish activities of virus-associated kinases, inhibitor-based strategies must take into account that the targeted kinases are not essential for the survival of the uninfected cells or, in the best scenario, for the survival of the infected cell itself. A more realistic anti-HIV-1 approach would rather rely on targeting either viral proteins used as substrates by virus-associated kinases or interactions directing incorporation into viral particles of cellular kinases indispensable for infectivity. The capacity of these strategies to reduce the acquisition of spontaneous mutations generated under the selective pressure exerted by conventional antivirals will require evaluation. These aspects will certainly merit attention when the knowledge on the function and packaging of HIV-1-associated kinases will have been significantly improved.

## Conclusions

In summary, a limited number of studies have uncovered the nature and the function of cellular kinases packaged into HIV-1 particles. While the experimental approaches developed remain imperfect, these studies have clearly shown that HIV-1-associated kinases are required for infectivity and act as key regulators at various steps of the replicative cycle, including nuclear shuttling of the preintegration complex and capsid assembly/disassembly. These functions are regulated through phosphorylation of HIV-1-encoded proteins. Given the variety of cellular kinases detected in viral particles, this family of proteins is expected to regulate additional steps of the HIV-1 life cycle. Analyzing their function will certainly provide new information regarding the biology of HIV-1. In addition, the capacity of HIV-1-associated kinases to target proteins in the newly infected cells has also been proposed although not formally demonstrated. This issue needs to be examined carefully to determine the contribution of virus-associated kinases in HIV-1 induced pathogenesis.

## Competing interests

The authors declare that they have no competing interests.

## Authors' contributions

NC and LB wrote the manuscript and made the figures. CG contributed to the manuscript writing and editing. All authors read and approved the final manuscript.
